# Electroresponsive and pH-Sensitive Hydrogel as Carrier for Controlled Chloramphenicol Release

**DOI:** 10.1021/acs.biomac.2c01442

**Published:** 2023-02-23

**Authors:** Leonor Resina, Karima El Hauadi, Jordi Sans, Teresa Esteves, Frederico Castelo Ferreira, Maria M. Pérez-Madrigal, Carlos Alemán

**Affiliations:** † Departament d’Enginyeria Química and Barcelona Research Center for Multiscale Science and Engineering, EEBE, Universitat Politècnica de Catalunya, C/Eduard Maristany 10-14, 08019 Barcelona, Spain; ‡ iBB − Institute for Bioengineering and Biosciences, Department of Bioengineering, Instituto Superior Técnico - Universidade de Lisboa, Avenida Rovisco Pais 1, 1049-001 Lisboa, Portugal; § Associate Laboratory i4HB−Institute for Health and Bioeconomy at Instituto Superior Técnico, Universidade de Lisboa, Avenida Rovisco Pais 1, 1049-001 Lisboa, Portugal; ∥ Institute for Bioengineering of Catalonia (IBEC), The Barcelona Institute of Science and Technology, Baldiri Reixac 10-12, 08028 Barcelona, Spain

## Abstract

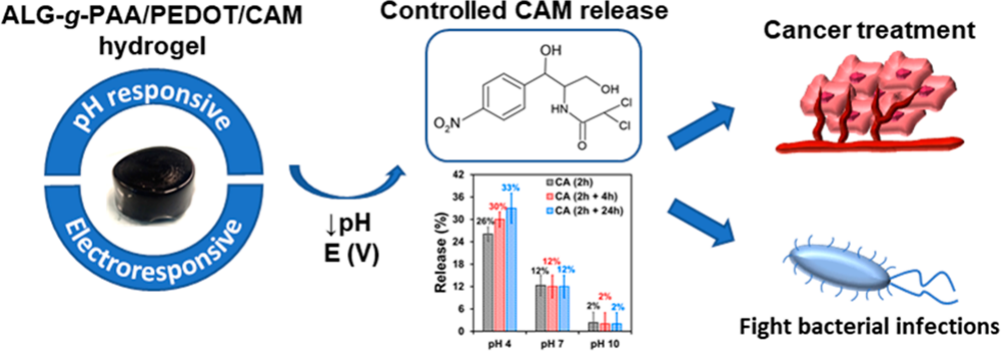

Multiresponsive hydrogels, which are smart soft materials that respond to more than one external stimulus, have emerged as powerful tools for biomedical applications, such as drug delivery. Within this context and with the aim of eliminating the systematic administration of antibiotics, special attention is being paid to the development of systems for controlled delivery of antibiotic for topical treatment of bacterial infections. In this work, an electro-chemo responsive hydrogel able to release chloramphenicol (CAM), a broad spectrum antibiotic also used for anticancer therapy, is proposed. This has been prepared by grafting poly(acrylic acid) (PAA) to sodium alginate (Alg) and in situ encapsulation of poly(3,4-ethylenedioxythiophene) nanoparticles loaded with CAM (PEDOT/CAM NPs), which were obtained by emulsion polymerization. Although the response to electrical stimuli of PEDOT was the main control for the release of CAM from PEDOT/CAM NPs, the release by passive diffusion had a relatively important contribution. Conversely, the passive release of antibiotic from the whole engineered hydrogel system, Alg-*g*-PAA/PEDOT/CAM, was negligible, whereas significant release was achieved under electrostimulation in an acid environment. Bacterial tests and assays with cancer cells demonstrated that the biological activity of CAM remained after release by electrical stimulation. Notably, the successful dual-response of the developed hydrogel to electrical stimuli and pH changes evidence the great prospect of this smart material in the biomedical field, as a tool to fight against bacterial infections and to provide local cancer treatment.

## Introduction

Recent advances in the field of drug delivery systems have shown the capability of releasing accurate amount of therapeutic agents or other bioactive substances at a specific location.^1−3^ This is particularly important for antibiotics since such drugs suffer several limitations that cannot be ignored as, for example, low accumulation and penetration in diseased cells/tissues, limited bioavailability of drugs, and off-target toxicity, among others.^4^ Furthermore, antibiotic overexposure predisposes to antibiotic resistance, which is a global public health problem.^5,6^ In order to avoid many of such problems, efforts have been focused on the development of smart systems for the release of antibiotics to the site of infection. These mainly consist of stimuli-responsive antibiotic delivery bioplatforms, which can release antibiotics in a controlled and timely fashion. These stimuli can either be exogenous (light,^7,8^ magnetism,^9,10^ ultrasound,^11,12^ and electrical^13,14^) or endogenous (pH,^15,16^ redox reactions,^17^ and enzymatic^18,19^).

Various types of antimicrobial release devices, such as hydrogels,^20,21^ nanoparticles (NPs),^22,23^ micro/nanofibers,^24,25^ and film-based reservoir devices,^26,27^ have been proposed. Among them, pulsatile antibiotic delivery systems using external electrical stimulation signals have drawn attention, as they allow repeatable and reliable drug release flux for therapeutic needs, thereby allowing remote control of local drug administration. Within this context, conducting polymers (CPs), which are organic materials with characteristics similar to those encountered in metals (i.e., good electrical and optical properties) and with the outstanding properties of conventional polymers (i.e., flexibility in processing, lightness of weight, and easiness in synthesis), play a major role.^13^ Among CPs, poly(3,4-ethylenedioxythiophene) (PEDOT) exhibits superior capacitive performance, high electrical conductivity, stability in aqueous media, and biocompatibility.^28−31^

PEDOT has been extensively used to load different types of drugs for subsequent controlled release through the application of different kinds of electrical stimuli.^14,32−36^ For example, anti-inflammatory dexamethasone was successfully released from loaded PEDOT films by applying cyclic voltammetry (CV) scans between −0.3 and 0.45 V,^32^ while anticancer botulin was delivered applying a constant potential of −0.5 V for 10 min.^33^ Curcumin, which displays a wide spectrum of medicinal properties, including antibacterial,^37^ was released from loaded PEDOT NPs using a constant potential at 0.50, −0.50, −1.00, or −1.25 V for 3 min.^34^ Instead, the release of chloramphenicol (CAM) from loaded PEDOT NPs was very slow, independent of these kinds of electrical stimuli.^14^ Despite such slowness, released CAM, which is a broad spectrum antibiotic that is effective against a variety of susceptible and serious bacterial infections,^38^ inhibited bacterial growth.^14^ A constant electrical potential was also successfully employed to release antibiotic ciprofloxacin from loaded PEDOT fibers^35^ and curcumin from loaded PEDOT hydrogels.^36^

Noteworthy, and most interesting, CAM, which has been reported to inhibit mitochondrial functions of eukaryotic cells,^39−41^ is also being considered as a potential option for cancer treatment.^42,43^ The metabolism of cancer cells, especially of cancer stem cells, is fundamentally regulated by an abundance of mitochondria compared to normal cells, including normal stem cells.^44^ Thus, in cancer cells, the low energy efficiency of the anaerobic metabolism is compensated with the presence of more mitochondria than in normal cells, which exhibit an aerobic metabolism. Accordingly, as part of an anticancer therapy, the utilization of CAM and other antibiotics that target cancer metabolisms reached great repercussion for its implications in clinical oncology.^44−47^ Indeed, in a recent study, Lamb et al.^44^ proved that CAM inhibits the formation of tumor stem cells, which are responsible for the metastasis by giving growth to new tumors.^48^

In this work, we go one step further, generating an electro-chemo-responsive system for the controlled release of CAM bearing in mind its dual biofunctionality. To engineer this multiresponsive system, we have harnessed the ability of PEDOT NPs to respond to electrical stimuli and assembled them into a hydrogel that responds to changes in pH. Although the extracellular pH at the end of the stationary phase of bacterial cell growth was found to be specific for each type of bacteria,^49,50^ most bacterial organisms grow best around pH values of 6.0–7.5, with some thriving in more acidic or alkaline conditions.^51^ Indeed, a pH range exists for which bacteria grow best, which comprises a minimum and maximum pH values to ensure growth, as well as an optimum pH. For instance, *Lactobacillus acidophilus*,^52^*Escherichia coli*,^53^ or *Staphylococcus aureus*(54) can survive in environments with pH as low as 4. On the other hand, tumors present a locally acidic environment that is now recognized as a tumor phenotype that drives cancer progression, causing tumor cells to become more invasive and lead to metastasis.^55^

Bearing the importance of the pH in mind, poly(acrylic acid) (PAA) hydrogels are known to exhibit reversible coil-to-globule conformational transitions at around pH 5, which are driven by the state of ionization of the carboxylic group. At low pH, PAA adopts a compact (but not fully collapsed) globular conformation (contracted hydrogel). Conversely, as the pH is increased, ionization occurs and the polymer expands into a fully solvated open coil conformation (expanded hydrogel).^56,57^ Herein, the abrupt contracted-to-expanded transition of PAA has been tuned by grafting PAA to sodium alginate (Alg) using *N*,*N*′-methylene-bis(acrylamide) (MBA) as cross-linker, the resulting hydrogel being denoted Alg-*g*-PAA. However, the ionization of the carboxylic groups with increasing pH has been employed to regulate the electrically induced release of CAM from loaded PEDOT NPs, the release decreasing drastically with increasing pH. It is worth noting that, although Alg hydrogels bear carboxylic acid groups, its direct use was avoided due to the fact that they do not experience volume changes associated with conformational transitions.^58^

## Methods

### Materials

To synthesize PEDOT NPs, sodium dodecyl benzenesulfonate (SDBS, technical grade) was used as surfactant, 3,4-ethylenedioxythiophene (EDOT; 97%) was the monomer, and ammonium persulfate (APS, (NH_4_)_2_S_2_O_8_; 98%)) acted as an oxidizing agent to initiate the polymerization. All such reactants were purchased from Sigma-Aldrich (U.S.A.). Chloramphenicol (CAM; 98%), which was loaded into PEDOT NPs, and phosphate buffered saline (PBS) solution, which was used as an electrolyte for electrochemical assays, were also purchased from Sigma-Aldrich.

For the Alg-*g*-PAA hydrogel synthesis, sodium alginate (Alg, low viscosity alginic acid sodium salt from brown algae), acrylic acid (AA; ≥99%) as comonomer, potassium persulfate (KPS, 99%) as oxidizing agent, and *N*,*N*′-methylene-bis(acrylamide) (MBA; 99%) as cross-linker were purchased from Sigma- Aldrich (U.S.A.). All reagents were used as received without further purification.

### Synthesis of PEDOT and PEDOT/CAM NPs

An emulsion polymerization was used to prepare the NPs. First, an SDBS surfactant solution (0.0815 g to 20 mL of milli-Q water, 9.3 mM) was prepared and kept at 40 °C and 750 rpm for 1 h. Then, EDOT monomer (88.8 μL, 32.2 mM) was added to the micellar solution. At the same time, 2.5 mL of water or 10 mg/mL of CAM solution in ethanol (0.025 g to 2.5 mL of ethanol) were added to the solution for the synthesis of PEDOT or PEDOT/CAM NPs, respectively. The mixtures were kept at 40 °C and 750 rpm for 1 h and, subsequently, an APS aqueous solution (0.456 g to 2.5 mL of milli-Q water, 0.8 M) was added. The reaction was kept at 40 °C and 750 rpm, protected from light, for 18 h. Purification of NPs and removal of unreacted reagents was achieved by three cycles of 40 min centrifugation at 4 °C and 11000 rpm, alternating with 20 min of sonication. The final product was then kept at 40 °C in an oven for 3 days until complete dryness. Afterward, the NPs were redispersed in milli-Q water by using a vortex and a sonication bath, to obtain 5 mg/mL of each NP category.

### Synthesis of Alg-*g*-PAA, Alg-*g*-PAA/PEDOT, and Alg-*g*-PAA/PEDOT/CAM Hydrogels

Alg-*g*-PAA hydrogel was prepared adapting the procedure reported by Thakur and Arotiba.^59^ For this purpose, Alg (0.5 g) was homogeneously dissolved at 50 °C in milli-Q water (19 mL). Then, 1.657 g of AA comonomer (1.21 M) and 0.025 g of MBA cross-linker (8.5 mM) were added to the Alg solution.

The same protocol was followed for preparing Alg-*g*-PAA hydrogel loaded with PEDOT NPs (Alg-*g*-PAA/PEDOT) or PEDOT/CAM NPs (Alg-*g*-PAA/PEDOT/CAM). The only difference was the addition of a mass of the corresponding NPs equal to 20% of the mass of Alg to the mixture containing Alg, AA, and MBA. This was followed by the incorporation of 0.026 g of KPS (5 mM) under stirring. The reaction mixtures were maintained at 70 °C for 1.5 h to complete the polymerization reaction. Afterward, the hydrogel was washed with acetone to remove unreacted reagents and stored at 4 °C until use.

### Characterization

Fourier transform infrared spectroscopy (FTIR) spectra were recorded on a FTIR Jasco 4100 spectrophotometer equipped with an attenuated total reflection accessory (top-plate) and a diamond crystal (Specac model MKII Golden Gate Heated Single Reflection Diamond ATR) connected to a computer with spectra manager software. For each sample, 32 scans were recorded between 4000 and 600 cm^–1^ with a resolution of 4 cm^–1^. To record the spectra of the NPs, 20 μL of PEDOT or PEDOT/CAM NPs aqueous dispersions (10 mg/mL) were dropped on aluminum foil and left overnight for solvent drying. FTIR spectra of Alg and AA comonomer were recorded using directly the reagent powder, while the Alg-*g*-PAA hydrogel was lyophilized for 3 days before analysis.

UV spectra were recorded using a Cary100 UV–vis spectrophotometer controlled by the UVProbe 2.31 software.

Scanning electron microscope (SEM) studies were performed in a Focused Ion Beam Zeiss Neon 40 scanning electron microscope operating at 5 kV. For visualization of NPs, samples were prepared by dropping 10 μL of PEDOT NPs (0.01 mg/mL) or PEDOT/CAM NPs (0.01 mg/mL) in suspension on aluminum foil. After being left overnight for solvent evaporation, the piece of foil was mounted on a double-sided adhesive carbon disc and coated with a thin carbon layer. For the estimation of NP size distributions, *n* = 100 was considered. In order to record SEM micrographs of Alg-*g*-PAA and Alg-*g*-PAA/PEDOT hydrogels, samples were first hydrated during 3 h, after which they were frozen for 10 min using liquid nitrogen. Then, the hydrogels were broken into two pieces (for the pores to be observed at the rupture zone) and lyophilized for 3 days. The resulting samples were then placed on a double-sided adhesive carbon disc and coated with a thin carbon layer. The size distribution of the pores was estimated considering *n* = 100.

High resolution transmission electron microscopy (TEM) was performed in a JEOL 2010F microscope equipped with a field emission electron source and operated at an accelerating voltage of 200 kV. The point-to-point resolution was 0.19 nm, and the resolution between lines was 0.14 nm. Samples were dispersed in an aqueous suspension using an ultrasonic bath, and a drop of the suspension was placed over a grid with holey-carbon film. Images were not filtered or treated by means of digital processing and they correspond to raw data.

Dynamic light scattering (DLS) and *z*-potential studies were performed using NanoBrook 90 Plus Zeta Potential Analyzer (Brookheaven Instruments Co., Blue Point Road Holtsville, NY, U.S.A.). Samples were resuspended in milli-Q water at a concentration of 0.01 mg/mL and placed into a cuvette of polystyrene with light pass of 1 cm to be analyzed at 25 °C using a scattering angle of 90°. The *z*-potential of PEDOT and PEDOT/CAM NPs was determined at pH 7.

The swelling response of the prepared hydrogels was studied as a function of the pH. For this purpose, Alg-*g*-PAA, Alg-*g*-PAA/PEDOT, and Alg-*g*-PAA/PEDOT/CAM lyophilized samples were cut in small pieces and immersed in 5 mL of 0.01 M PBS at pH 4 (adjusted using a hydrochloric acid solution), 7, and 10 (adjusted using a sodium hydroxide solution), under agitation at 80 rpm and 37 °C. The weight of the wet (i.e., swollen) hydrogels was measured at different times (0, 0.5, 1, 2, 4, 6, 24, 48, and 72 h) to calculate the swelling ratio (SR) after the surface moisture of the hydrogel was removed:
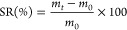
1where *m*_*t*_ is the mass of the swollen sample at time *t* (hydrated in 0.01 M PBS at pH 4, 7, or 10 for *t* hours) and *m*_0_ is the mass of the samples at time 0 h (i.e., as synthesized). All experiments were conducted considering three repetitions (*n* = 3).

The equilibrium water content (EWC) was calculated using eq 2:
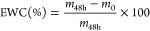
2where *m*_48h_ refers to the mass of the wet hydrogel (hydrated in 0.01 M PBS at pH 4, 7, or 10 for 48 h) after the surface moisture was removed. Finally, the gel fraction (GF) was defined as
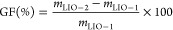
3where *m*_LIO-1_ is the mass after lyophilizing the hydrogel one time (i.e., *m*_LIO-1_ = *m*_0_) and *m*_LIO-2_ is the mass of the hydrogel after lyophilizing, rehydrating for 24 h in 0.01 M PBS to remove the remaining soluble fraction, and lyophilizing again (*n* = 3).

All rheology studies were performed on an Anton Parr MCR 302 rheometer fitted with a parallel plate configuration (diameter of 20 mm) at 25 °C and using a solvent trap. Hydrogels were prepared and submitted to the condition tested (either 24 h in PBS at pH 4, 7 or 10; or 2 h under the CA electrical stimuli at pH 4 followed by 24h in PBS at pH 4), after which samples were cut with dimensions of 2 cm in diameter and 0.5 cm in thickness. Before testing, the upper plate was carefully lowered to a plate separation of 1 mm, the hydrogel was trimmed and the measurement was started. Frequency sweeps were carried out applying a constant strain of 1%, while the frequency was ramped logarithmically from 0.1 to 100 rad/s. Meanwhile, amplitude sweeps were conducted applying a constant frequency of 1 Hz (6.28 rad/s), while the strain was ramped logarithmically from 0.1% to 5000%. All measurements were repeated in triplicate, and representative/averaged charts are shown.

### CAM Release from PEDOT/CAM NPs

To trigger the release of CAM from PEDOT/CAM NPs, two different electric stimuli, CV and chronoamperometry (CA), were evaluated. CV and CA cycles were applied using an Autolab PGSTAT302N and NOVA software. For CV cycles, the initial and final potential were −0.50 V, and the reversal potential was +0.80 V, while the scan rate was 0.1 V/s. Each CA cycle consisted of the application of a potential of 0.60 V for 100 s, followed by an interruption (i.e., 0.00 V) for 5 min, and, subsequently, the application of a potential of −0.6 V for 100 s, followed by another interruption for 5 min. The CA cycles were repeatedly applied for 2 h.

A drop of 20 μL of PEDOT (control) or PEDOT/CAM NPs (5 mg/mL) in suspension was deposited on the surface of a screen-printed carbon electrode (SPCE) and left to dry. Then, the coated SPCE was immersed in a cell containing 3 mL solution of 0.01 M PBS, as an electrolyte, to record the CVs and CAs. The PBS solution was collected afterward.

The concentration of CAM in the release medium (*n* = 3) was determined using the absorbance of the release medium at 278 nm and the corresponding absorbance–CAM concentration calibration plot in 0.01 M PBS.

### CAM Release from Alg-*g*-PAA/PEDOT/CAM

The controlled release of the antibiotic from the Alg-*g*-PAA/PEDOT/CAM hydrogel was performed applying CA cycles as electrical stimulus. Assays were conducted using an Autolab PGSTAT302N and NOVA software in a three-electrode cell. The electrochemical setup consisted of a Pt counter electrode, an Ag|AgCl reference electrode, and the Alg-*g*-PAA/PEDOT/CAM hydrogel as working electrode. The release medium was 5 mL of 0.01 M PBS solution at pH 4, 7, and 10. CA cycles identical to those described above for PEDOT/CAM NPs were applied for 2 h to stimulate the release from the hydrogel. The medium was collected right after such time, and 4 and 24 h after, and analyzed by UV–vis spectroscopy.

### Bactericidal Activity

The bactericidal activity of the loaded CAM was tested with *Escherichia coli* (*E. coli*), *Streptococcus sanguinis* (*S. sanguinis*), and *Streptococcus mutans* (*S. mutans*) using the inhibition zone method. First, 1 mL of an overnight culture (grown for 16 h) was added to 5 mL of the Lysogeny broth (LB) medium. Bacteria were seeded on LB agar plates and the samples, which included hydrogel pieces of Alg-*g*-PAA/PEDOT/CAM with two CAM loadings (33 and 66 μg/mL), as well as paper discs impregnated with 20 μL of release solutions, were deposited on top. Negative controls consisted of 20 μL release media from passive diffusion of Alg-*g*-PAA/PEDOT and Alg-*g*-PAA) hydrogels. The positive control was a disc impregnated with 20 μL of a CAM solution in water at 66 μg/mL. The effect of CAM on bacterial growth (i.e., antibacterial performance) was evaluated after incubation at 37 °C for 24 h.

### Cell Viability Assays

Cell viability assays were performed with Vero and HeLa cell lines using the MTT assay. Briefly, cells were seeded at a concentration of 1 × 10^4^ cells/well in 96-well plates and incubated overnight at 37 °C and 5% CO_2_. Cells were then exposed to a dilution series of CAM-containing released media from Alg-*g*-PAA/PEDOT/CAM hydrogels by electrical stimulation (CA), as well as to free CAM (positive control; initial concentration 33 μg/mL), for 24 h at 37 °C and 5% CO_2_. The negative control was cell culture medium without CAM. After the incubation period, the MTT labeling reagent was added to each well and incubated for 3 h at 37 °C and 5% CO_2_, followed by the solubilizing agent (dimethyl sulfoxide). Finally, absorbance was measured at 570 nm, and the percentage of viable cells relative to untreated control was determined. The results were expressed as average value ± standard deviation (*n* = 3).

## Results and Discussion

### Preparation and Characterization of the Nanoparticles

**Scheme 1:**
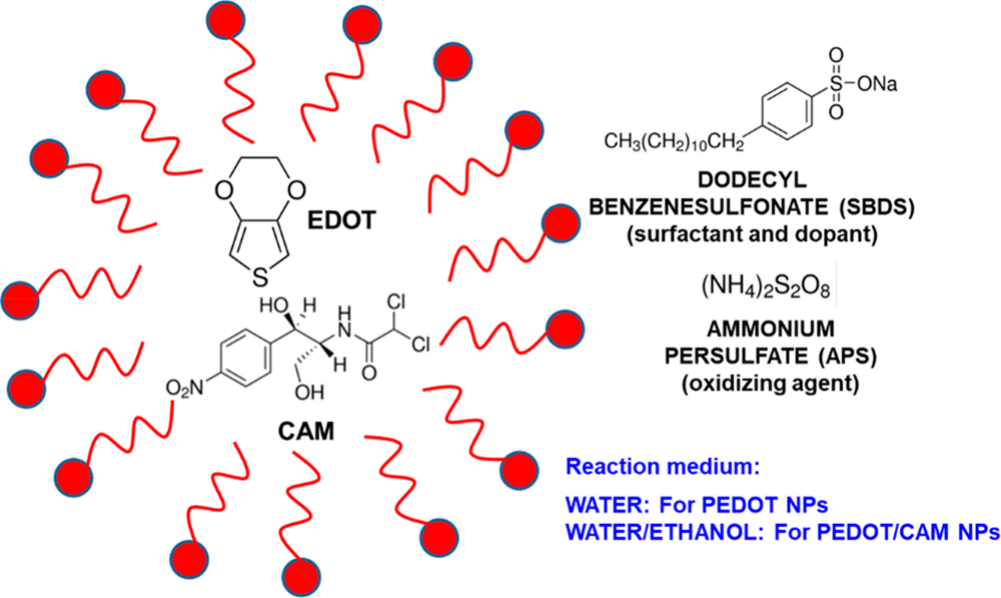
Sketch Illustrating the Emulsion Polymerization of PEDOT and PEDOT/CAM NPs

**Figure 1:**
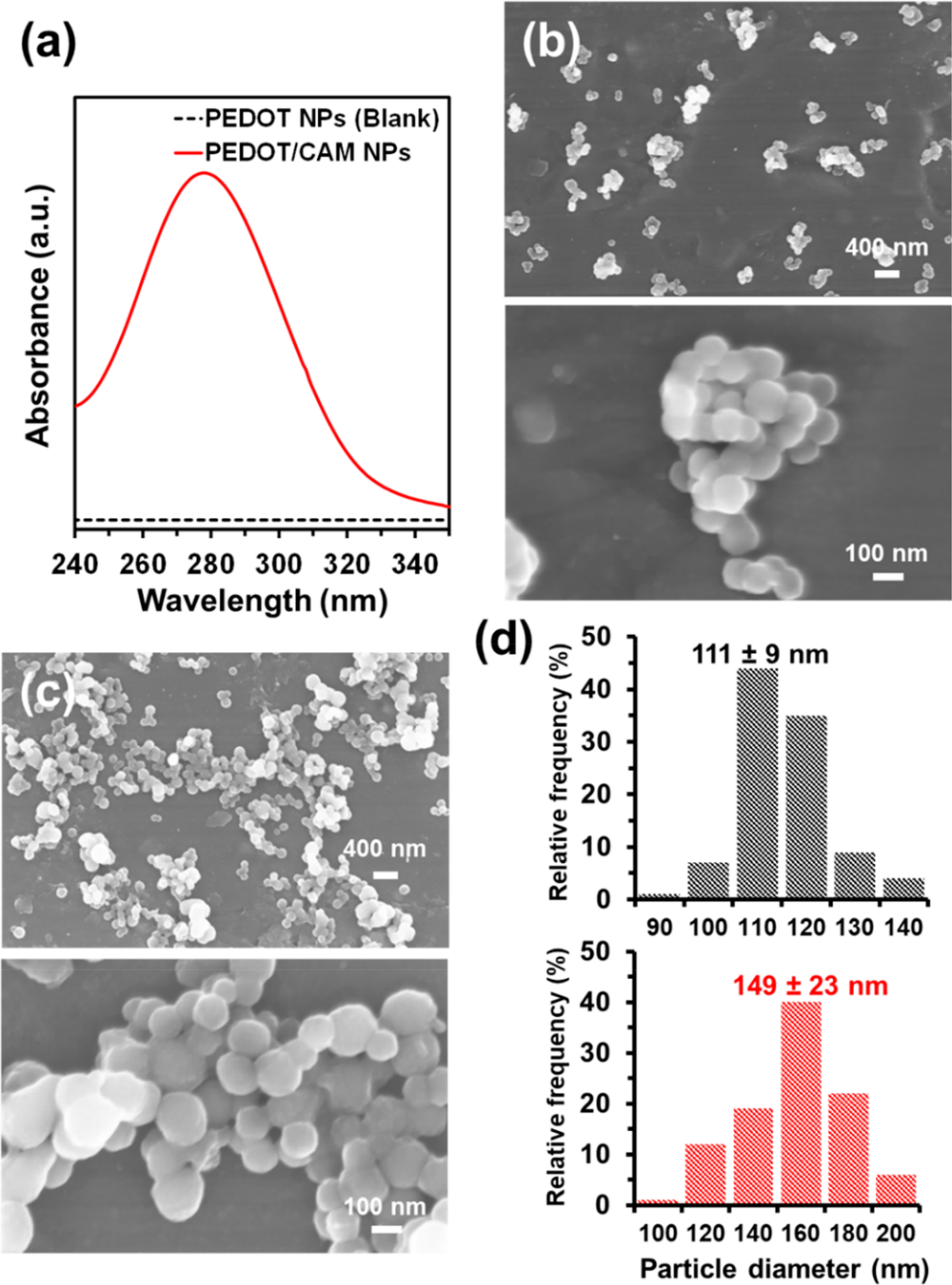
(a) UV–vis spectra of CAM extracted from PEDOT/CAM NPs using ethanol (solid red line) compared to the blank (PEDOT NPs; black dashed line). (b, c) SEM micrographs (top, 16k× magnification; bottom, 80k× magnification) of (b) PEDOT and (c) PEDOT/CAM NPs. (d) Size distribution histogram (*n* = 100) of PEDOT (top) and PEDOT/CAM NPs (bottom).

PEDOT and PEDOT/CAM NPs were prepared by emulsion polymerization, the drug being loaded in situ during the synthesis (Scheme 1). The FTIR spectra of PEDOT and PEDOT/CAM NPs, which are shown in Figure S1, display the characteristic peaks of PEDOT that correspond to the C–O–C vibrations (1215 and 1066 cm^–1^), CH_2_ stretching (2924 cm^–1^), C=C in the thiophene ring (1715 cm^–1^), C–C inter-ring stretching and C–S–C vibrations (835 and 687 cm^–1^). Unfortunately, the FTIR spectrum of free CAM, which is included in Figure S1, shows many peaks overlapping the characteristic peaks found for unloaded PEDOT, precluding the identification of the antibiotic in PEDOT/CAM NPs. However, the successful loading of CAM was confirmed by UV–vis spectroscopy. For this purpose, suspensions of PEDOT/CAM and PEDOT (blank) NPs were incubated in ethanol for 2 weeks to completely extract the antibiotic and then, UV–vis spectra were recorded for the resulting solutions once the solid residues were eliminated by centrifugation. Figure 1a shows that the solution coming from PEDOT/CAM NPs exhibits the characteristic absorption peak at 278 nm, while no peak was obtained from the blank solution derived from PEDOT NPs.

The absorbance–CAM concentration plot displayed in Figure S2 was used as calibration curve to quantify the loading capacity (LC, in %) of PEDOT NPs, expressed as mass of CAM loaded in PEDOT/CAM NPs (*m*_loaded_) relative to the total mass of the NPs (*m*_NPs_):
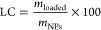
4It is worth noting that *m*_loaded_ corresponds to the mass of CAM initially incorporated to the solution used for the synthesis of PEDOT/CAM (0.025 g) minus the mass of CAM remaining free in the solution after the synthesis of PEDOT/CAM. Details about the quantification of the loaded CAM are provided in the Supporting Information (SI).

The LC estimated for the PEDOT/CAM NPs prepared in this work was 14.3% ± 2.5%, which is slightly higher than the one reported for PEDOT/CAM NPs prepared using dodecylbenzenesulfonic acid (DBSA), rather than SDBS, as stabilizer and dopant agent (LC: 11.9% ± 1.3%).^14^ Furthermore, the LC achieved in this work with SDBS micelles was higher than those obtained for PEDOT/curcumin (CUR) and PEDOT/piperine (PIP) NPs, which were also prepared using DBSA micellar solutions, by 8.4% and 6.0%, respectively.^34^

The morphology of PEDOT and PEDOT/CAM NPs was studied by SEM (Figure 1b,c). Unloaded PEDOT NPs show the typical spherical morphology (Figure 1b) with an effective diameter of 111 ± 9 nm (Figure 1d), as determined by measuring the diameter of 100 NPs randomly selected, which was similar to that obtained for PEDOT NPs synthesized using DBSA (96 ± 16 nm).^34^ The size of the NPs increased to 149 ± 23 nm (Figure 1d), similarly to what was also observed for PEDOT NPs loaded with anticancer pentapeptides.^60^

**Figure 2:**
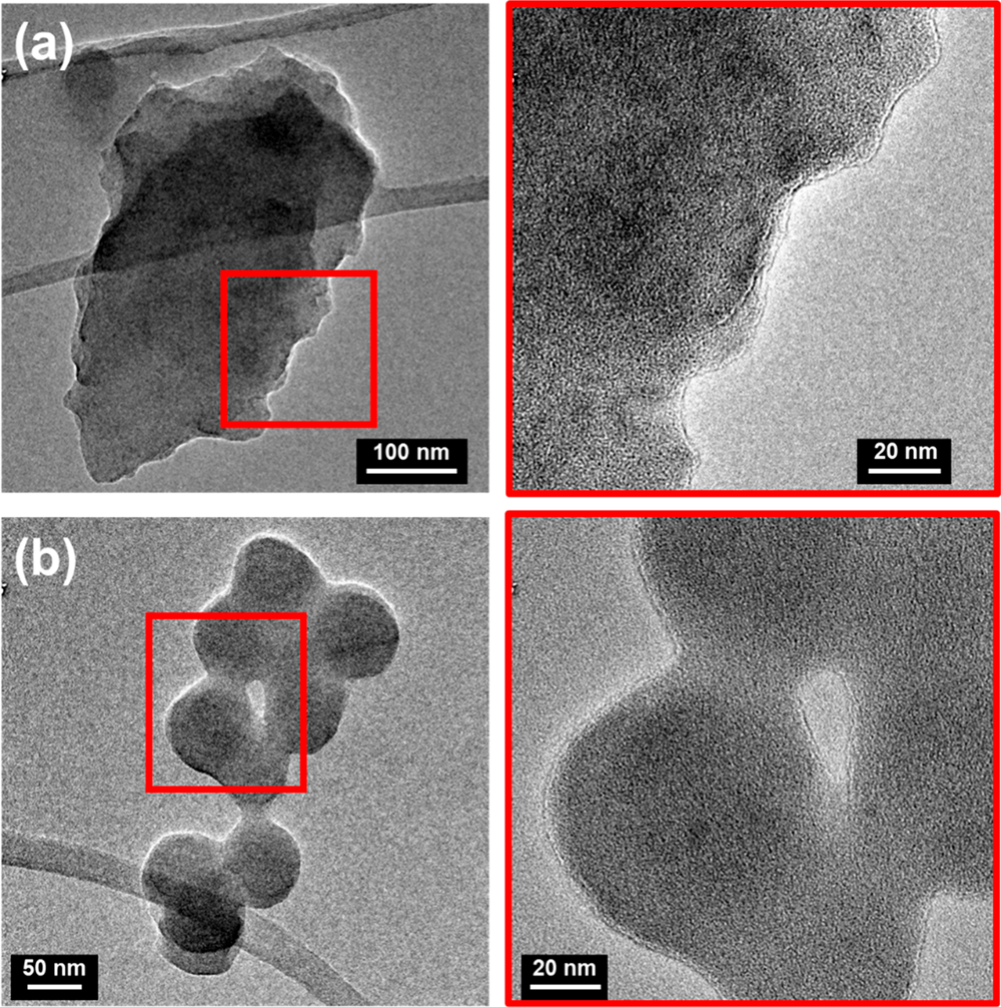
High resolution TEM micrographs of (a) PEDOT and (b) PEDOT/CAM NPs.

Morphological phenomena were less clear in high resolution TEM micrographs due to the aggregation artifacts produced during the preparation of the samples, TEM results evidenced that the CP chains adopted an amorphous structure in both PEDOT and PEDOT/CAM NPs (Figure 2).

DLS measurements confirmed the effect of the antibiotic on the average size of the NPs, the values obtained using this optical technique being 157 ± 27 and 268 ± 10 nm for PEDOT and PEDOT/CAM NPs, respectively. The diameters determined by DLS were higher than the ones estimated by SEM, which was attributed to the fact that the former technique provides the hydrodynamic size of the NPs in solution (i.e., water and SDBS molecules in the hydrodynamic shell are included), while the latter one gives the size of dry NPs. However, the ratio between the diameters of loaded NPs to unloaded NPs, was very similar for DLS and SEM measurements (1.7 and 1.6, respectively).

The *z*-potential, which is a measure of the effective electric charge on the NPs surface, was determined to examine the tendency toward aggregation of PEDOT and PEDOT/CAM NPs. Both unloaded and loaded NPs exhibited negative *z*-potential values (−30 ± 2 and −29 ± 3 mV, respectively, at 0.01 mg/mL), which are consistent with a high degree of suspension stability and, therefore, a low tendency to agglomerate. As expected, the *z*-potential increased in suspensions with increasing PEDOT/CAM concentrations (e.g., −7 ± 3 and −5 ± 3 mV for 0.1 and 0.5 mg/mL suspensions, respectively).

### Electrostimulated Release of CAM from PEDOT/CAM Nanoparticles

Antibiotic release studies from PEDOT/CAM NPs were conducted in PBS considering two different kinds of electrical stimuli. The first consisted in applying potentiodynamic CV cycles with electrical potential scans ranging from −0.50 V (initial and final potential) to +0.80 V (reversal potential) at a scan rate of 0.1 V/s, which represents 140 CV cycles per hour. The second kind of electrical stimulus was the application of potentiostatic CA cycles, each cycle comprising the application of a constant voltage of +0.60 V for 100 s and, after an interruption of 5 min, a constant voltage of −0.60 V for 100 s, followed by another interruption of 5 min (i.e., 4.5 CA cycles per hour). The released CAM was quantified by measuring the absorbance at 278 nm and using the calibration plot obtained for CAM in PBS (Figure S3). More specifically, the percentage of released CAM was defined using eq 5:
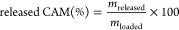
5where *m*_released_ indicates the mass of CAM in the release medium after 2 or 4 h and *m*_loaded_ is the mass of CAM loaded in PEDOT/CAM NPs after synthesis.

**Figure 3:**
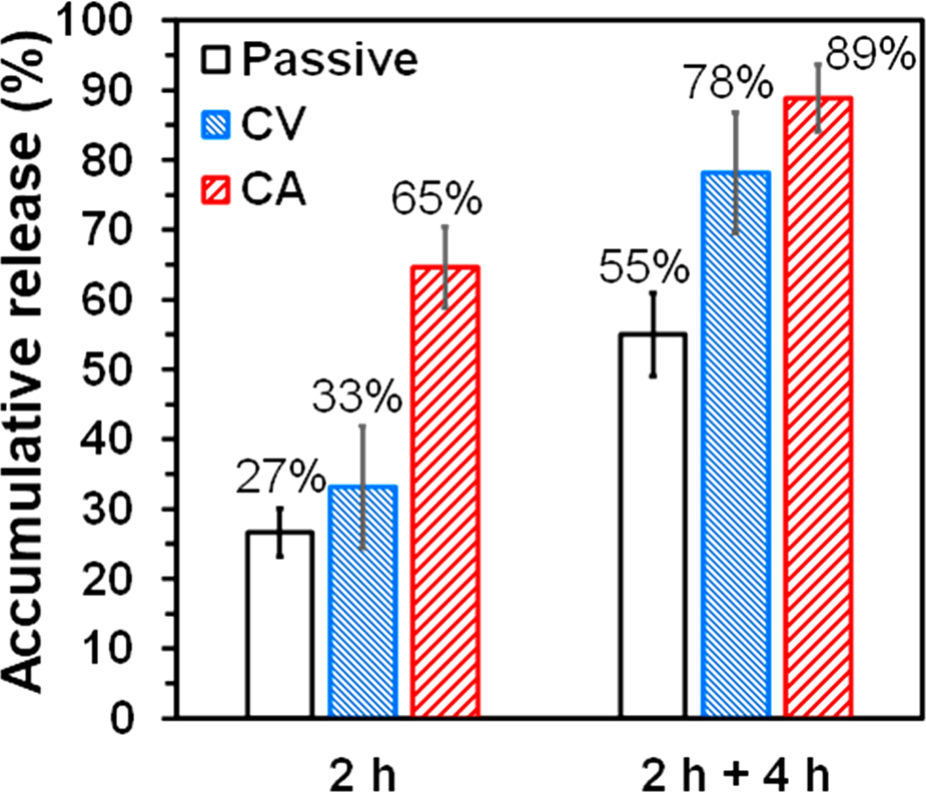
Accumulative release of CAM at different times as observed at pH 7 by passive diffusion and applying CV and CA electrical stimuli.

Figure 3 compares the passive release of CAM from PEDOT/CAM NPs with that induced by electrostimulation using CV or CA for 2 h (i.e., 2 h in Figure 3 refers to 280 CV or 9 CA cycles) and 4 h after each electrostimulation regime was finished (i.e., 2 h + 4 h in Figure 3 refers to 280 CV or 9 CA cycles +4 h of passive diffusion). As it can be seen, the passive release, which occurred by the diffusion of CAM molecules across the NPs matrix due to a concentration gradient, was relatively fast, increasing around 14% per hour. Electrostimulation resulted in a faster release rate, this response being more evident for CA than for CV, especially at the shortest time (i.e., 33% ± 9% and 65% ± 6% for CV and CA after 2h, respectively). Moreover, it should be emphasized that the release achieved after 9 CA cycles plus 4 h of passive diffusion reached 89% ± 5%, which is much higher than the one observed by simple passive diffusion (55% ± 6%).

The success of the CA stimulus was attributed to the fact that the alternate application of positive and negative potentials favored the swelling and shrinking of the NP matrix through the entrance (positive electrical potential) and escape (negative electrical potential) of solvated counter-anions, thus, enhancing CAM release with respect to nonstimulated passive diffusion.

### Preparation and Characterization of Hydrogels

**Scheme 2:**
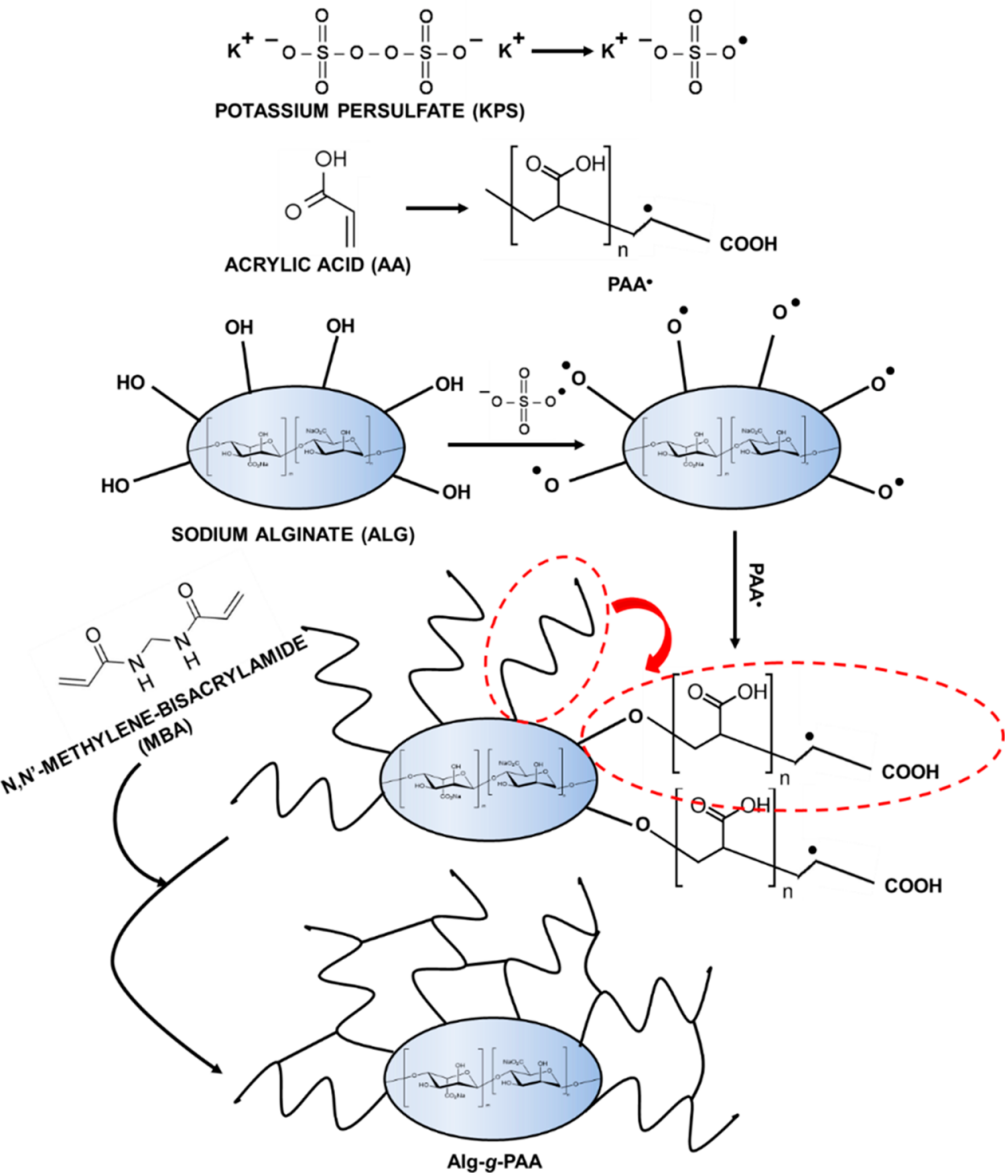
Sketch Illustrating the Graft Polymerization Method Used to Prepare Alg-*g*-PAA

Alg-*g*-PAA hydrogel was prepared by aqueous polymerization, grafting AA monomer onto Alg, and using MBA and KPS as cross-linker and oxidizing agent, respectively (Scheme 2).^59^ The incorporation of PEDOT and PEDOT/CAM NPs into Alg-*g*-PAA to produce Alg-*g*-PAA/PEDOT and Alg-*g*-PAA/PEDOT/CAM, respectively, was performed in situ, adding the corresponding NPs to the reaction mixture before to introduce the oxidizing agent.

**Figure 4:**
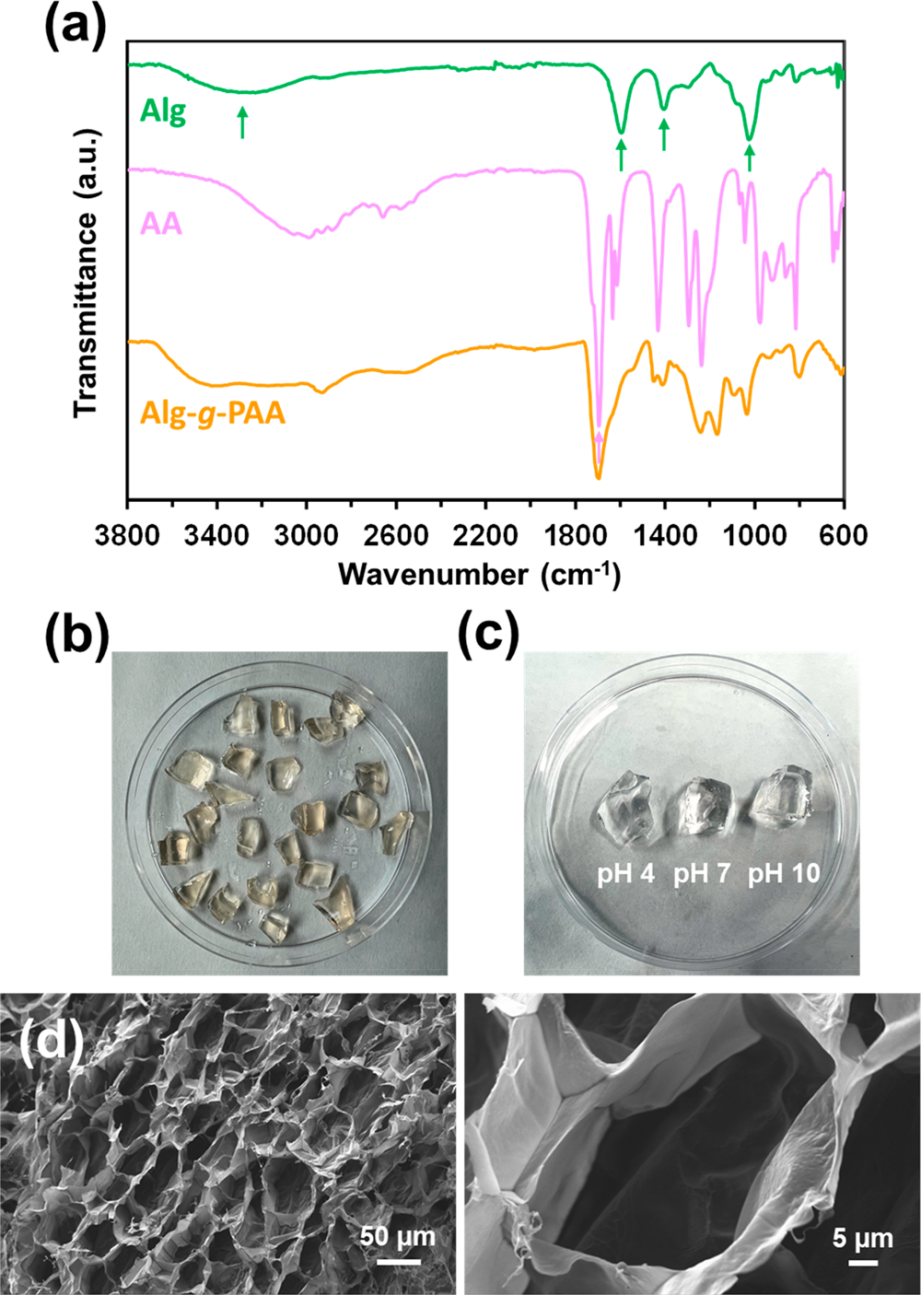
(a) FTIR spectra of Alg, AA, and Alg-*g*-PAA hydrogel after lyophilization. (b, c) Photographs of Alg-*g*-PAA (b) as synthesized and (c) after 48 h of hydration in 0.01 M PBS under stirring (80 rpm) at 37 °C and pH 4, 7, or 10. (d) SEM micrographs of Alg-*g*-PAA (left, 200× magnification; right, 1.5k× magnification).

The FTIR spectrum of Alg-*g*-PAA hydrogel is compared in Figure 4a with those of Alg and AA comonomer. The spectrum of Alg-*g*-PAA confirms the success of the grafting process, as it contains the characteristic bands of both Alg and AA. More specifically, Alg-*g*-PAA and Alg spectra exhibit the broad band at ∼3430 cm^–1^ (O–H stretching), the intense bands at 1595 and 1408 cm^–1^ assigned to carbonyl (C=O asymmetric and symmetric stretching, respectively), and the peak at 1026 cm^–1^ (C–O–C stretching).^61^ The Alg-*g*-PAA spectrum also shows an intense peak at 1701 cm^–1^ assigned to the C=O stretching of the AA comonomer.

Although as prepared Alg-*g*-PAA displays a semitransparent yellowish color arising from Alg (Figure 4b), its hydration not only induces the expected expansion of volume, but also a change toward a transparent appearance, independent of the pH (Figure 4c). SEM micrographs of the lyophilized hydrogel indicate that the hydrogel presents an interconnected porous structure with the typical honeycomb morphology (Figure 4d). Pores exhibit thin walls and a distorted round shape, with the average size being 44 ± 9 μm (*n* = 100).

**Figure 5:**
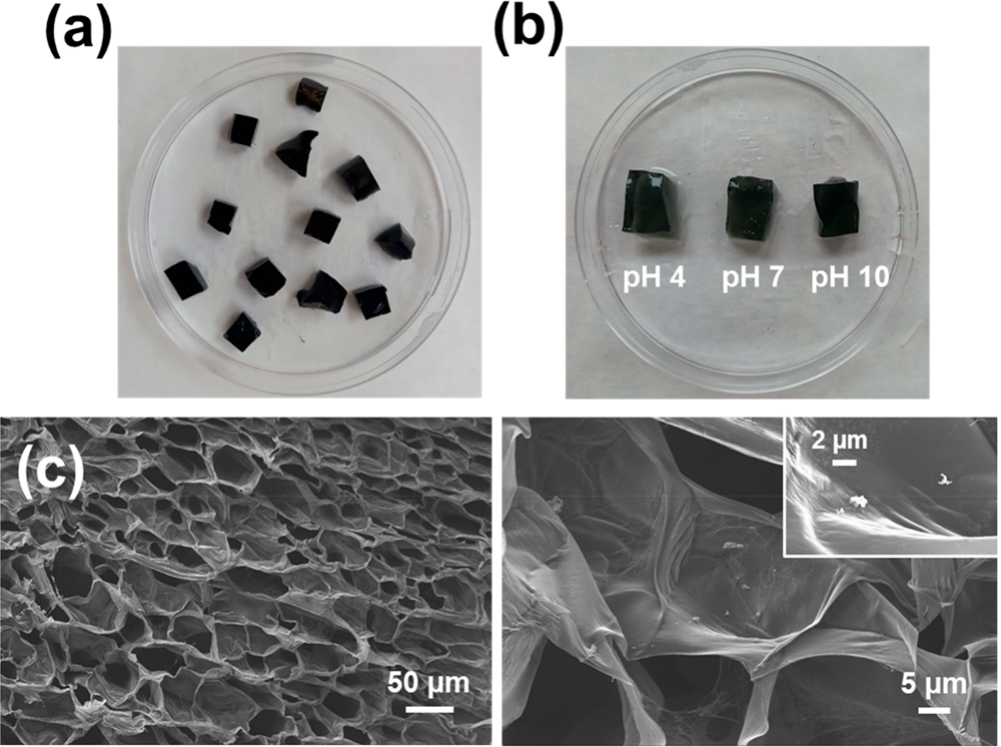
(a, b) Photographs of Alg-*g*-PAA/PEDOT (a) as synthesized and (b) after 48 h of hydration in PBS under stirring (80 rpm) at 37 °C and pH 4, 7, or 10. (d) SEM micrographs of Alg-*g*-PAA/PEDOT (left, 200× magnification; right, 1.5k× magnification).

The successful loading of PEDOT NPs on the Alg-*g*-PAA hydrogel was evidenced by a change from the yellowish color to the characteristic dark blue color of PEDOT, as can be seen in Figure 5a. The volume expansion of Alg-*g*-PAA/PEDOT observed upon hydration is very high at the three studied pH values (Figure 5b), as occurred for the hydrogel without PEDOT NPs. SEM micrographs of Alg-*g*-PAA/PEDOT (Figure 5c) reflect an interconnected structure similar to that described for Alg-*g*-PAA, the average pore size being practically identical in both cases (42 ± 9 and 44 ± 9 μm for Alg-*g*-PAA/PEDOT and Alg-*g*-PAA, respectively). However, magnified micrographs (Figure 5c, right) show submicrometric clusters of PEDOT NPs spread on the pores of the Alg-*g*-PAA/PEDOT hydrogels and also inside the walls of the pores.

**Figure 6:**
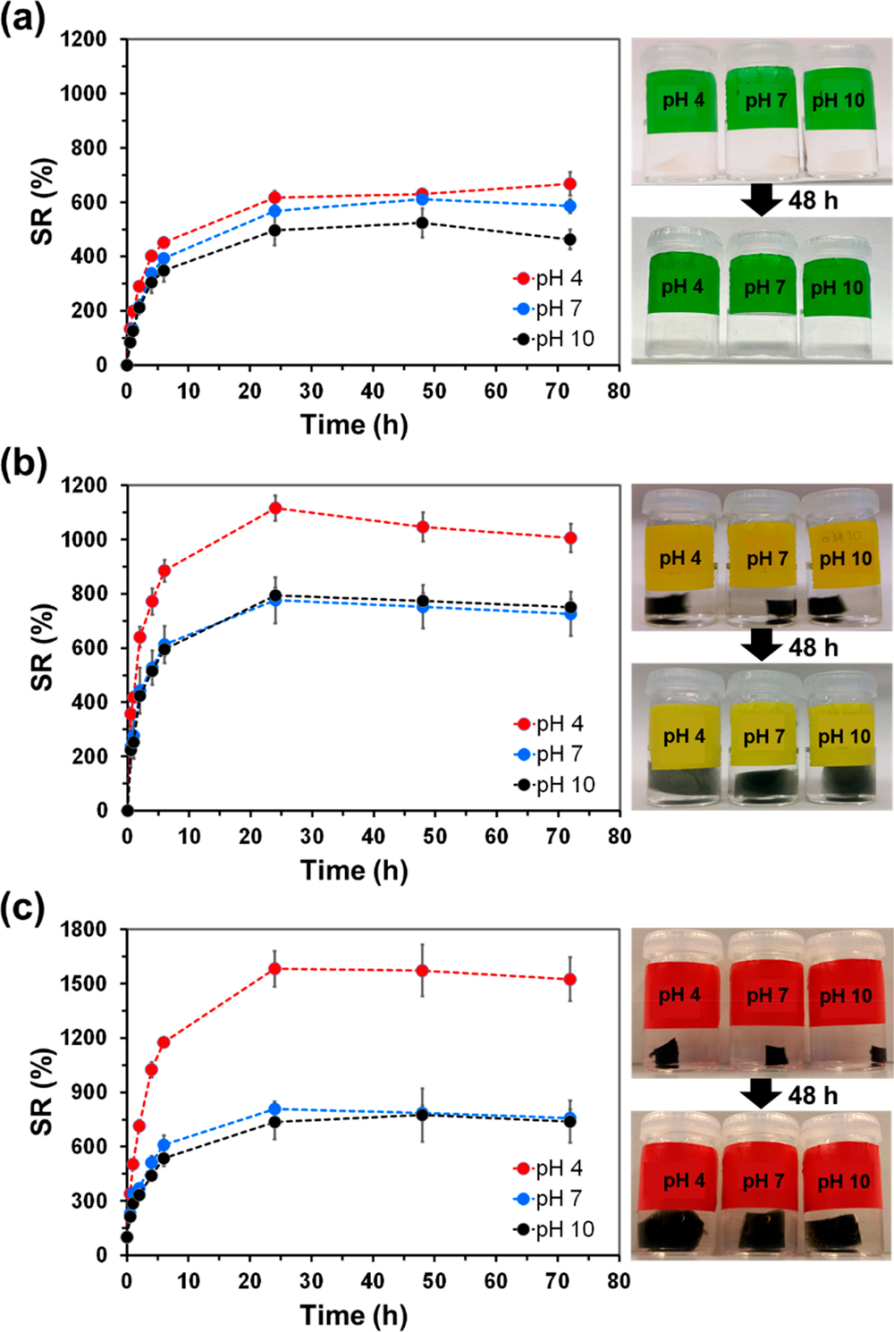
Temporal evolution of the SR (*n* = 3; left) and photographs of the hydrogels as immersed and after 48 h of immersion in PBS solution (right) for (a) Alg-*g*-PAA; (b) Alg-*g*-PAA/PEDOT; and (c) Alg-*g*-PAA/PEDOT/CAM.

In order to examine the pH response of the prepared hydrogels, both Alg-*g*-PAA and Alg-*g*-PAA/PEDOT dry samples were cut in small pieces and immersed in 5 mL of 0.01 M PBS at pH 4, 7, and 10 under 80 rpm and 37 °C. Visual inspection (naked eye) of the hydrogel photographs as immersed and after 48 h of hydration (Figure 6) evidenced their high swelling capacity. In order to quantify such observation, the temporal evolution of the swelling ratio (SR; eq 1) was determined by weighting the swollen hydrogels at different times considering different pH conditions. For Alg-*g*-PAA hydrogel, hydrogen bonding interactions between the protonated carboxylic acid groups (from both Alg and PAA) were expected to be very abundant at the acid pH, thus, reducing the swelling capacity of the hydrogel; while at neutral and basic pHs ionized carboxylate groups were expected to generate repulsive electrostatic interactions within the hydrogel network, allowing very high SRs.^62^ Conversely, Figure 6a revealed a behavior completely different from that expected. More specifically, similar SRs were observed at pH 4 and 7, while the swelling obtained at pH 10 was slightly lower. This has been attributed to the shielding effect of the hydrated Na^+^ ions from the media on the carboxylate groups of the hydrogel, which results in a significant reduction of the repulsive interactions at pH 7 and 10, affecting noticeably the swelling capacity. Thus, due to their higher strength,^63^ the interactions of charged ions with water are more stabilizing than hydrogen bonds between the protonated carboxylic acid groups.

For Alg-*g*-PAA/PEDOT, the SR at acid pH was clearly higher than at neutral and basic pH, while the latter two exhibited very similar curves (Figure 6b). Furthermore, the SR of Alg-*g*-PAA/PEDOT at acid pH is significantly higher than that of Alg-*g*-PAA, independently of the time. Similarly, the same trend was detected for Alg-*g*-PAA/PEDOT/CAM (Figure 6c). Such enhanced swelling behavior has been attributed to two main aspects: (i) the presence of PEDOT NPs disrupt hydrogen bonding interactions, thus allowing the expansion of the hydrogel network at low pH; and (ii) the SDBS surfactant molecules contained in the PEDOT NPs increase the negative charge of the network and, hence, the repulsive forces, which further expanded the hydrogel network. The most relevant advantage of this behavior (i.e., enhanced expansion of the hydrogel with PEDOT NPs at acid pH) favors the utilization of such system for the controlled delivery of CAM in the acid environment of the tumors’ sites, while also displaying antibacterial effect to fight infections.

On the other hand, the equilibrium water content (EWC) of Alg-*g*-PAA, given by eq 2, is practically independent of the pH: 86.3% ± 0.3%, 85.9% ± 0.3 and 83.9% ± 1.4% at pH 4, 7, and 10, respectively. Conversely, not only are the EWC values obtained for Alg-*g*-PAA/PEDOT higher than those of Alg-*g*-PAA, but also exhibit some pH dependence: 91.3% ± 0.4%, 88.1% ± 0.3, and 85.7% ± 0.3% at pH 4, 7, and 10, respectively. These values, which are consistent with the expansion of the PEDOT-containing hydrogel at acid pH, are similar to those obtained for Alg-*g*-PAA/PEDOT/CAM (93.5% ± 1.1%, 87.2% ± 0.7, and 86.8% ± 2.3% at pH 4, 7, and 10, respectively). The gel fraction (GF; eq 3) of Alg-*g*-PAA, Alg-*g*-PAA/PEDOT, and Alg-*g*-PAA/PEDOT/CAM is 97% ± 1%, 96% ± 2%, and 94% ± 1%, respectively, indicating that the cross-linking efficiency in such hydrogels is very high.

**Figure 7:**
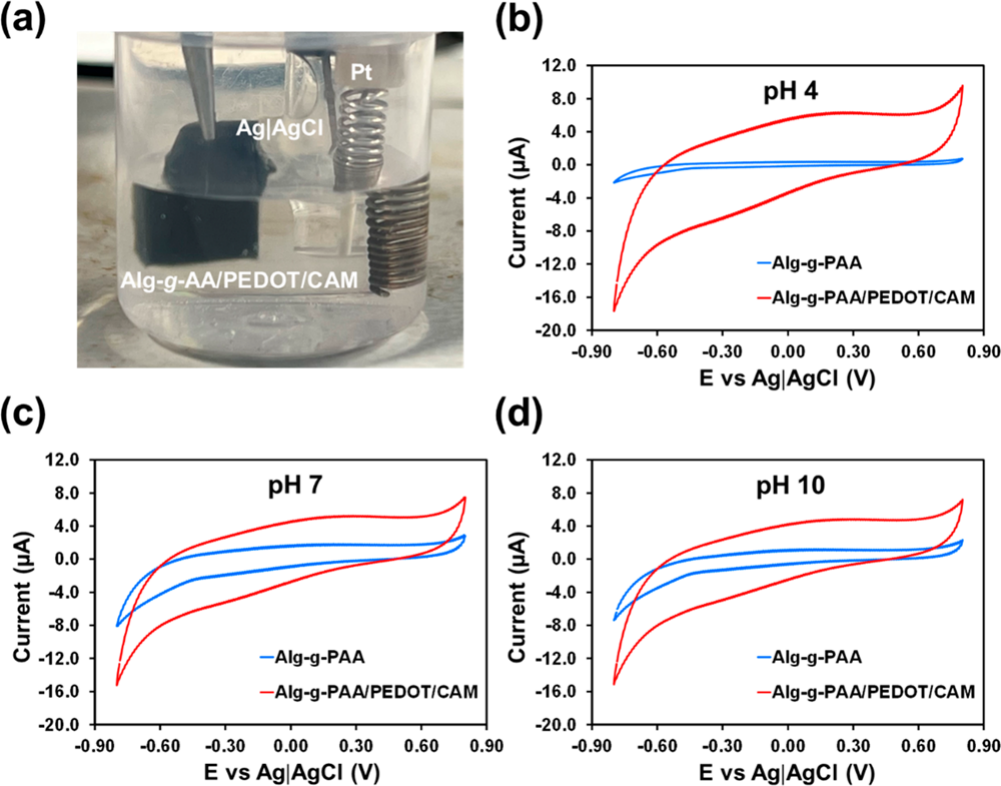
(a) Set-up used for electrochemical measures. (b–d) Cyclic voltammograms recorded for Alg-*g*-PAA and Alg-*g*-PAA/PEDOT/CAM at (b) pH 4, (c) 7, and (d) 10.

To compare the electrochemical responses of Alg-*g*-PAA and Alg-*g*-PAA/PEDOT/CAM, both hydrogels were studied by CV using the setup displayed in Figure 7a. As it can be seen, the hydrogels were directly used as working electrodes, while the counter and reference electrodes consisted of a Pt wire and an Ag|AgCl electrode. The cyclic voltammograms recorded in 0.01 M PBS at different pHs are compared in Figure 7b–d.

As expected, the electrochemical activity of Alg-*g*-PAA, which is proportional to the area of the cyclic voltammogram, is enhanced by loaded electroactive PEDOT NPs. This feature was found to depend on the pH (Figure 7b–d), the increment of electrochemical activity being higher at the acid pH. Quantitative comparison between Alg-*g*-PAA and Alg-*g*-PAA/PEDOT/CAM reveals that the CP increases the electrochemical activity by 1482%, 172%, and 277% at pH 4, 7, and 10, respectively. Such behavior correlated well with the swelling response observed earlier: a more open structure (i.e., expanded hydrogel network) was obtained at pH 4, which promoted the entrance and escape of ions during the redox process of PEDOT NPs during electrical stimulation.

**Figure 8:**
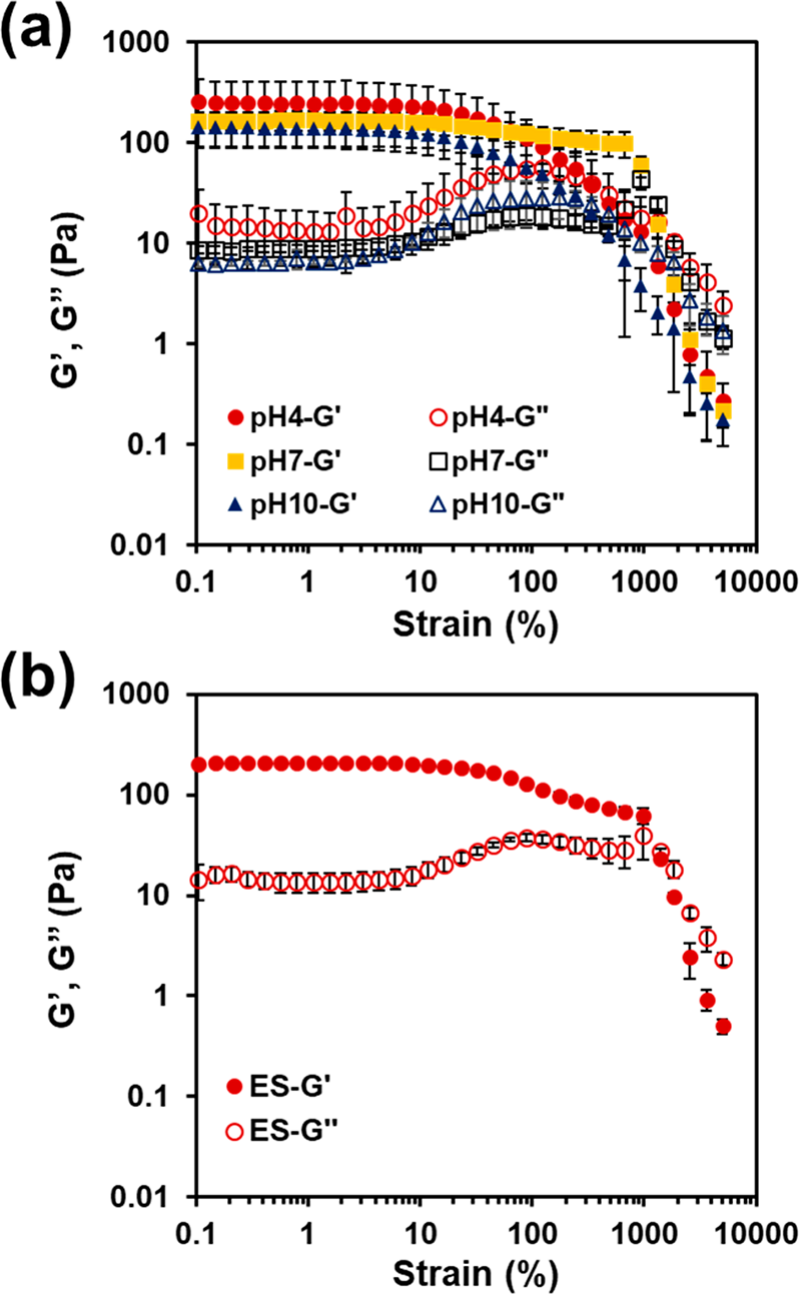
Rheological data for Alg-*g*-PAA/PEDOT/CAM hydrogels recorded under amplitude sweep (at 1 Hz) from 0.1 to 5000% after (a) being immersed in PBS at different pH values for 24 h and (b) after applying the CA electrical stimulus for 2 h (pH 4), followed by 24 h in PBS at pH 4. Error bars: SD with *n* = 3.

The viscoelastic properties of as prepared Alg-*g*-PAA/PEDOT/CAM hydrogels were determined by rheological characterization. The storage modulus (*G*′), which accounts for the material’s ability to store energy elastically under shear, was monitored by running both amplitude and frequency sweeps (Figure S4). Specifically, from the amplitude sweep, *G*′ was determined to be 317 ± 75 Pa (at 10% strain). The viscoelastic performance of the hydrogels remained stable up to 100% strain, when *G*′ values started to decline and, ultimately, yielded at strain values higher than 100% and reaching *G*′′ > *G*′ at 1300%. After immersion in PBS for 24 h, the *G*′ values of the hydrogels determined at 12% strain (amplitude sweep) decreased down to 222 ± 134, 158 ± 36, and 120 ± 46 Pa for pH 4, 7, and 10, respectively, on account of the swelling process, which produced a softer material (Figure 8a).

In terms of yielding, *G*′ values for samples kept at pH 4 and 10 started to yield at lower strain values, and the crossover between *G*′ and *G*′′ occurred between 300 and 400% strain. In contrast, the response of the samples kept at pH 7 was more similar to that of the as prepared system. Hence, swelling did modify to some extent the viscoelastic performance of Alg-*g*-PAA/PEDOT/CAM hydrogels, being more noticeably for pH 4 and 10. On the other hand, the hydrogel submitted to the electrical stimuli described in the Methods section (at pH 4) displayed a *G*′ value of 200 ± 10 Pa (at 12% strain), as seen earlier, which indicated that the electrochemical process had little effect on the hydrogel viscoelastic response (Figure 8b).

### CAM Release from Alg-*g*-PAA/PEDOT/CAM

**Figure 9:**
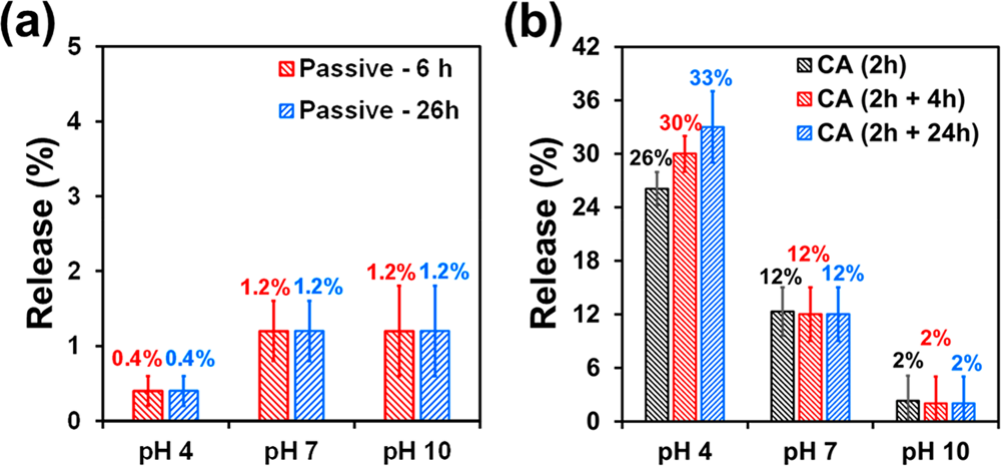
Release of CAM from Alg-*g*-PAA/PEDOT/CAM at different pHs (*n* = 3) as observed by (a) passive diffusion after 6 and 26 h and (b) just after applying the CA electrical stimuli, which took 2 h, and both 4 and 24 h later.

The passive and electrostimulated release of CAM from Alg-*g*-PAA/PEDOT/CAM was studied at different pHs. Electrostimulation was performed by applying CA cycles identical to those used for PEDOT NPs for 2 h (i.e., a total of 9 CA cycles). Analysis of the drug delivered in absence of stimuli indicated that, after 24 h, most of the drug remains in the carrier, independently of the pH (Figure 9a). Indeed, the amount of CAM passively released, which does not increase with the time of immersion in the medium, is around 1% only. This result represents a drastic reduction with respect to PEDOT/CAM NPs (Figure 3), for which the passive release reached a value of around 55% after only 6 h, evidencing that CAM does not only interact with PEDOT chains, but also with water molecules. Conversely, the very slow passive release observed when PEDOT/CAM NPs are loaded into the Alg-*g*-PAA has been attributed to the strength of the interactions formed by the drug and the polar groups of the hydrogel. Thus, such interactions are apparently much stronger than those it could form with water molecules, preventing the diffusion of the CAM molecules through the hydrogel matrix to exit the medium.

On the other hand, the CAM release from Alg-*g*-PAA/PEDOT/CAM increased significantly upon electrostimulation (Figure 9b). This feature is fully consistent with results obtained for PEDOT/CAM NPs (Figure 3), which confirms that PEDOT/CAM NPs preserve their response to CA cycles when embedded in the hydrogel. Furthermore, the release increased upon decreasing pH, reaching values of 30%, 12%, and 2% at pH 4, 7, and 10, respectively, after 6 h (i.e., 2 h of electrostimulation + 4 h). Moreover, for pH 4, the release increases to 33% after 26 h (i.e., 2 h of electrostimulation + 24 h). Comparison of these results with those obtained by passive diffusion for Alg-*g*-PAA/PEDOT/CAM and by electrostimulation for PEDOT/CAM NPs suggests that the chronoamperometric-induced CAM release from Alg-*g*-PAA/PEDOT/CAM was driven by the content of water inside the hydrogel (i.e., water entropy-driven mechanism). Thus, CA cycles induced the release from the loaded NPs entrapped in the hydrogel, while the competing interactions between the released CAM molecules and either the water molecules and polar groups of the Alg-*g*-PAA matrix were affected by pH. It is worth noting that the hydrogel SR was found to be much higher at acid pH than at neutral pH, which in turn was higher than at basic pH (Figure 7b). Accordingly, the abundance of CAM molecules interacting with water increased with decreasing pH, explaining the variation of the released antibiotic with the pH that occurred by diffusion of the CAM molecules that were not interacting with the hydrogel matrix.

Comparison of the release profiles displayed in Figure 9 with those reported for conventional hydrogels reveals that Alg-*g*-PAA/PEDOT/CAM presents significant advantages in terms of control and targeting. For example, the release from CAM-loaded Alg-based hydrogels, which are chemo-responsive to the calcium ion concentration, was recently studied by different authors.^64,65^ In deionized water, an initial fast release followed by a sustained rate of release was observed without applying external stimulus (i.e., passive release).^64^ Indeed, complete (cumulative release of 100%) release was achieved in around 3 h only. However, this effect was slightly delayed (i.e., cumulative release of 40–60% release in 3 h) by enhancing the interactions with the drug through the loading of cellulose nanocrystals into the hydrogel,^64^ or by increasing the concentration of calcium ions to increase the cross-linking.^65^ Thus, the incorporation of PAA and PEDOT NPs allows to drastically reduce the passive release and, at the same time, to provide pH-selective response to electrical stimuli.

The need for materials with both broad utility and greater application specificity is ever-present. In the case of drug delivery applications, hydrogels with specific, tunable and reversible responses to environmental stimuli are known for decades to be excellent candidates as drug vehicle.^66^ Current drug delivery research is evolving from biomimetic materials that are responsive to the host environment to smart materials that respond to multiple stimuli, allowing to better dose and targeting control release.^67^ This feature is particularly relevant when the released drug an anticancer medication, which usually exhibit a high toxicity profile. Considering the local acidic pH environment of tumors present a locally acidic environment, Alg-*g*-PAA/PEDOT/CAM is a sophisticated smart material that fulfils all such requirements. The chemo- and electro-response of Alg-*g*-PAA/PEDOT/CAM, which favors the release of CAM under electrostimulation in an acid environment, enables a more controlled and efficient release with a hierarchical targeting strategy that was not achieved using single-responsive carriers.^14,21,22^ Moreover, the proposed system is expected to work in the same way when drugs similar in size and polarity are used instead of CAM.

### Antibacterial Tests

**Figure 10:**
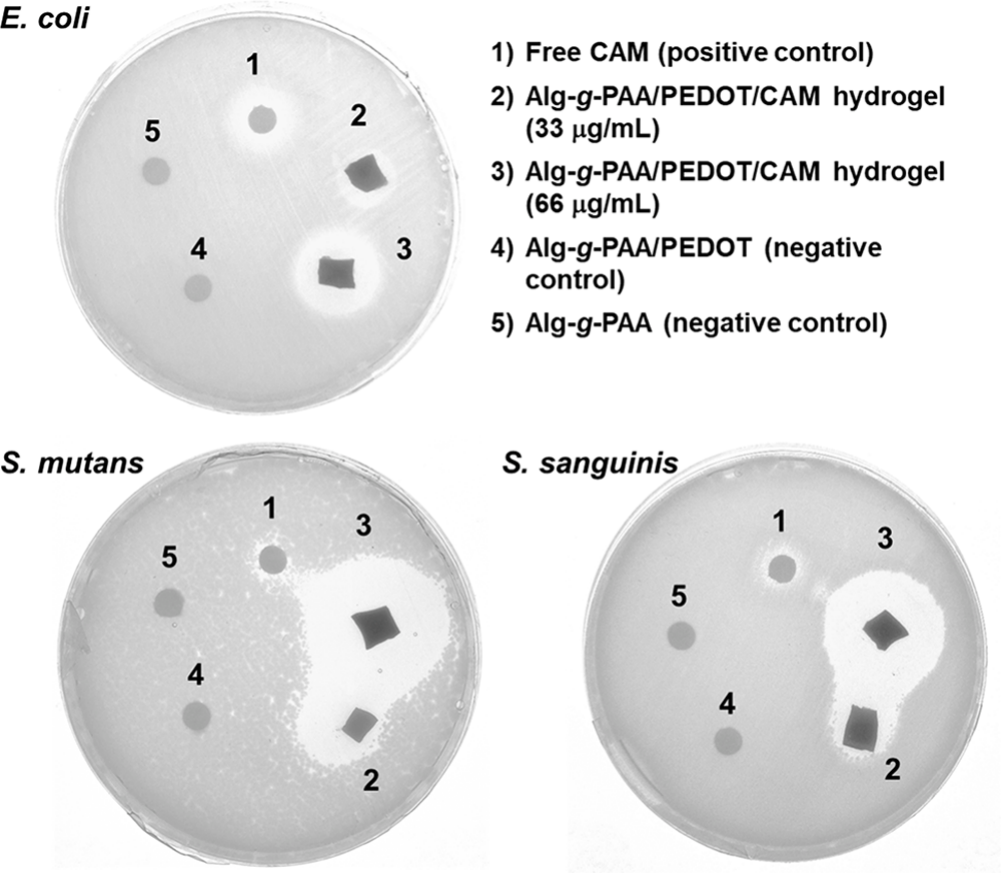
Bactericidal activity against *E. coli*, *S. sanguinis*, and *S. mutans* of free CAM (positive control; 1), Alg-*g*-PAA/PEDOT/CAM hydrogels with two drug loading concentrations (33 and 66 μg/mL; 2 and 3), 20 μL of the release medium after passive diffusion from Alg-*g*-PAA/PEDOT hydrogel (without CAM; 4), and 20 μL of the release medium after passive diffusion from Alg-*g*-PAA hydrogels (without CAM nor PEDOT NPs; 5). Inhibition halos observed using the disk diffusion method.

Results from the antibacterial activity of released CAM, which was tested against Gram-negative (*E. coli*) and Gram-positive bacteria (*S. sanguinis* and *S. mutans*), are shown in Figure 10.

The activity of CAM was not altered after being introduced in the Alg-*g*-PAA/PEDOT/CAM hydrogel. Thus, the release of the drug from Alg-*g*-PAA/PEDOT/CAM by passive diffusion was effective for inhibiting bacterial growth, which is a concentration dependent mechanism. Free CAM (positive control) also hindered bacteria growth, even though, in this case, the inhibition zone was smaller probably as a consequence of the lower dose deposited onto the disk (i.e., 20 μL at 66 μg/mL). As it was expected, no antibacterial activity was detected for release media samples (20 μL) derived from the passive diffusion of Alg-*g*-PAA/PEDOT and blank Alg-*g*-PAA hydrogels (both without CAM).

### Anticancer Activity

**Figure 11:**
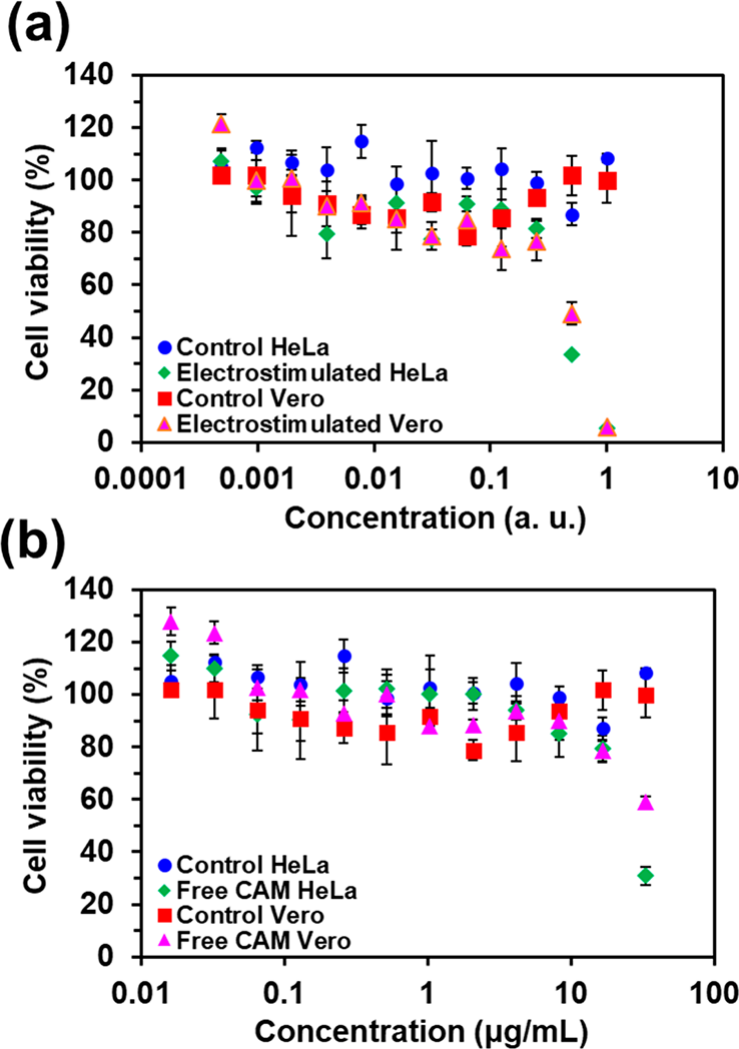
(a) Cell viability values for HeLa and Vero cell lines, after being exposed for 24 h to CAM released from Alg-*g*-PAA/PEDOT/CAM hydrogels by applying the CA electrical stimulus or (b) exposed to free CAM. Error bars indicate the standard deviation (*n* = 3).

The anticancer activity of released CAM was examined using HeLa cells (an immortalized cell line derived from cervical cancer cells), as well as Vero cells (kidney tissue derived from a normal, adult African green monkey). Specifically, cell viability was determined for cells after being exposed to CAM released by electrostimulation from Alg-*g*-PAA/PEDOT/CAM hydrogels (Figure 11a), as well as to free CAM (Figure 11b). The dilution series was achieved by successive 1:2 dilutions of the initial concentrations (i.e., 33 μg/mL for free CAM). The concentration in Figure 11a is expressed in arbitrary unit, where a concentration of 1 au refers to the initial CAM concentration in the release media after electrostimulation.

In general, cell viability decreases with increasing drug concentration, independently of the source of CAM (i.e., free or released); however, HeLa cell are more sensitive to the presence of CAM (Figure 11b), being the cell viability higher for Vero cells (59%) than for HeLa (31%) cells at 33 μg/mL. For lower drug concentrations, cell viability is higher than 80%, regardless of the cell line. This response is also observed for CAM released from Alg-*g*-PAA/PEDOT/CAM hydrogels by electrostimulation (Figure 11a). Interestingly, the initial drug concentration in the release medium might be higher than 33 μg/mL, as initially calculated, thus reducing cell viability for both cell lines. Overall, these features confirm that the potential anticancer activity of CAM was not altered during the encapsulation process or the release by electrostimulation. Next steps in device design should include a careful optimization to further adjust CAM dosage.

## Conclusions

Alg-*g*-PAA/PEDOT/CAM hydrogels were prepared by incorporating spherical PEDOT/CAM NPs of average diameter 149 ± 23 nm, which are electroresponsive, into the pH responsive Alg-*g*-PAA hydrogel during its synthesis. The properties of Alg-*g*-PAA/PEDOT/CAM, which were fully characterized using different techniques, evidenced that the SR depends on the pH and that the hydrogel is conductive. CAM-release tests from PEDOT/CAM NPs, which showed a LC of 14.3% ± 2.5%, revealed a relatively fast passive release rate (i.e., around 14% per hour) that increased by applying CV or, especially, CA stimuli. Conversely, CAM-release assays from Alg-*g*-PAA/PEDOT/CAM showed that the passive release was negligible, regardless of the pH. This response has been attributed to the formation of specific interactions between CAM molecules released from the embedded PEDOT/CAM NPs and the polar groups of the Alg-*g*-PAA matrix. When CA electrostimuli were applied to Alg-*g*-PAA/PEDOT/CAM, the amount of CAM molecules released from the NPs to the hydrogel increased and, concomitantly, the diffusion out of the hydrogel increased with the SR (i.e., with decreasing pH). Antibacterial tests and cell viability assays proved that the biological activity of CAM was not altered during the loading and release processes.

Considering the bioactivity of CAM, the proposed conducting hydrogel is of particular interest for the treatment of cancer, as well as regulated inhibition of bacterial infections, avoiding the increased antibiotic resistance as patients undergo systemic treatments. Results show that Alg-*g*-PAA/PEDOT/CAM hydrogel allows electro-chemo controlled release of CAM, a broad spectrum antibiotic, which occurs when the pH of the environment is acid and PEDOT/CAM NPs are electrostimulated. Further studies on this bioplatform could lead to an optimization of different variables, including the control of stimulation parameters (e.g., duration of the electric stimuli, magnitude of the potential, etc.), as well as a more precise understanding of the pH effect by considering different environments with different acidities (e.g., pH 4.5, 5.0, 5.5, 6.0, and 6.5). Additionally, this system also has the potential to release other antibiotics or drugs, or a combination thereof, to environments of specific requirements where the electro-chemo response can be custom exploited.

## Supporting Information Available

Experimental methods for quantification of the loading capacity, FTIR spectra, and calibration plots (PDF)
