# Supplementary material for: Electroresponsive and pH-Sensitive Hydrogel as Carrier for Controlled Chloramphenicol Release
Source: Biomacromolecules. 2023 Feb 23;24(3):1432–44. doi: 10.1021/acs.biomac.2c01442 (PMC10889591; doi:10.1021/acs.biomac.2c01442)
Supplement: Supplementary file 1 — bm2c01442_si_001.pdf [file bm2c01442_si_001.pdf]

SUPPORTING INFORMATION

Electro-Responsive and pH-Sensitive Hydrogel as Carrier for Controlled Chloramphenicol Release

Leonor Resina,[a,b,c] Karima El Hauadi,[a] Jordi Sans,[a] Teresa Esteves,[b,c] Frederico Castelo Ferreira,[b,c] Maria M. Pérez-Madrigal,[a,*] and Carlos Alemán[a,d,*]

[a] Departament d'Enginyeria Química and Barcelona Research Center for Multiscale Science and Engineering, EEBE, Universitat Politècnica de Catalunya, C/ Eduard Maristany 10-14, 08019, Barcelona, Spain

[b] iBB – Institute for Bioengineering and Biosciences, Department of Bioengineering, Instituto Superior Técnico - Universidade de Lisboa, Avenida Rovisco Pais 1, 1049-001 Lisboa, Portugal

[c] Associate Laboratory i4HB—Institute for Health and Bioeconomy at Instituto Superior Técnico, Universidade de Lisboa, Avenida Rovisco Pais 1, 1049-001 Lisboa, Portugal

[d] Institute for Bioengineering of Catalonia (IBEC), The Barcelona Institute of Science and Technology, Baldiri Reixac 10-12, 08028 Barcelona Spain

* Correspondence to m.mar.perez@upc.edu and carlos.aleman@upc.edu

METHODS

Loading capacity (LC, in %). For the quantification of the LC, which was obtained using Eq 4, the absorbance-CAM concentration plot displayed in Figure S2 was determined. For such purpose, 10, 20 and 50 μL of PEDOT/CAM NPs solutions at a concentration of 5 mg/mL were incubated in ethanol absolute to a total of 1 mL and left at 4 ºC for 2 weeks to promote the full unstimulated release of CAM from the NPs. The solutions were centrifuged at 11000 rpm and the supernatants were analyzed by UV-visible spectroscopy.

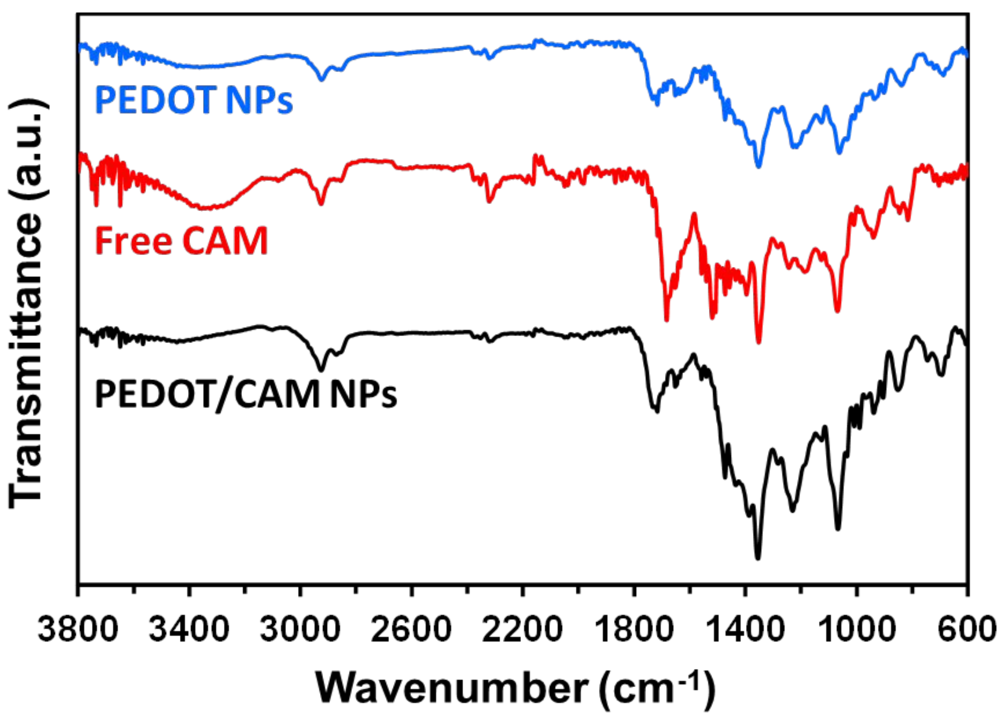

Figure S1. FTIR spectra of PEDOT NPs, free CAM and and PEDOT/CAM NPs.

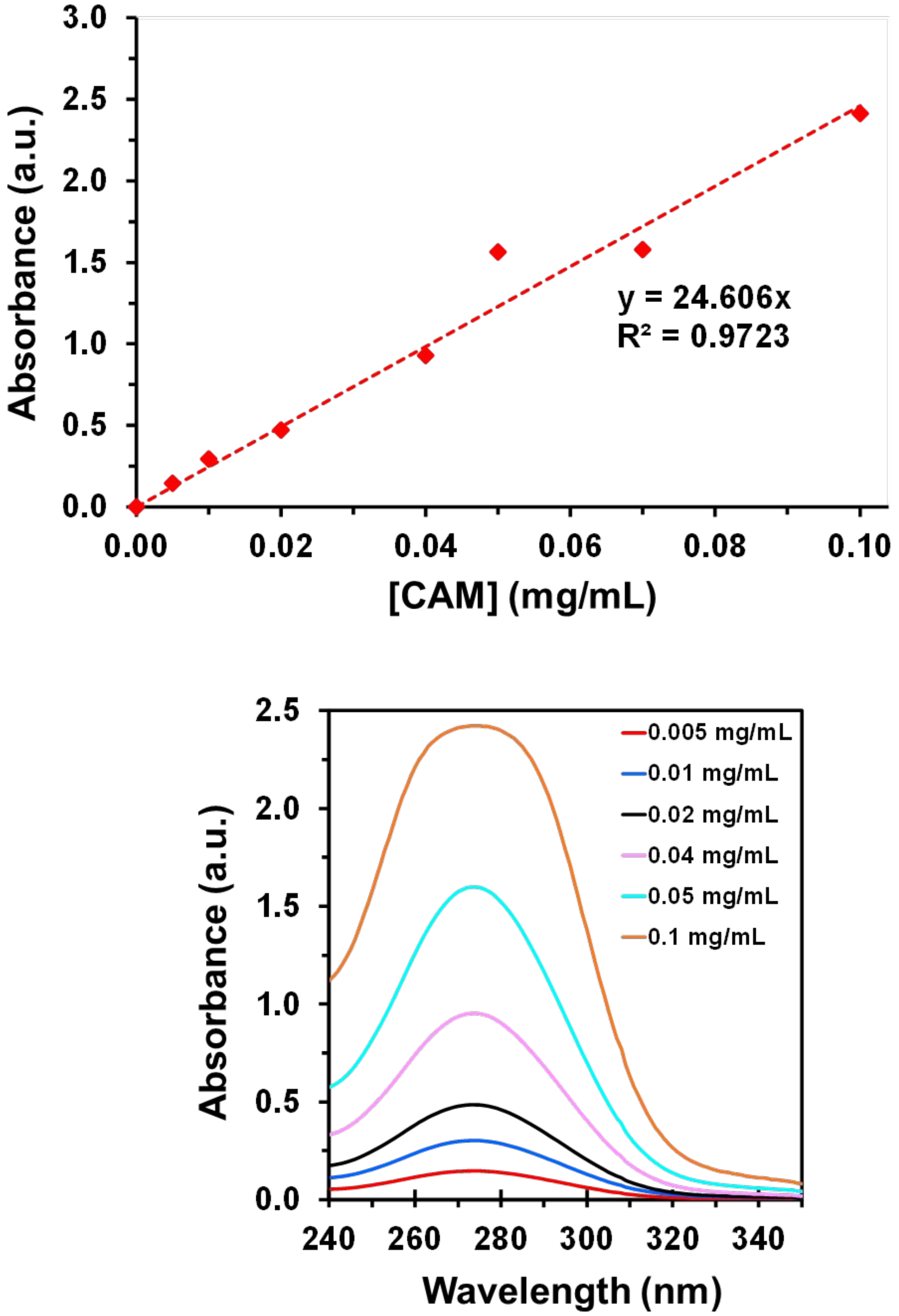

Figure S2. Calibration curve (absorbance-CAM concentration plot) for CAM in PBS. Bar errors are lower than the size of the symbols.

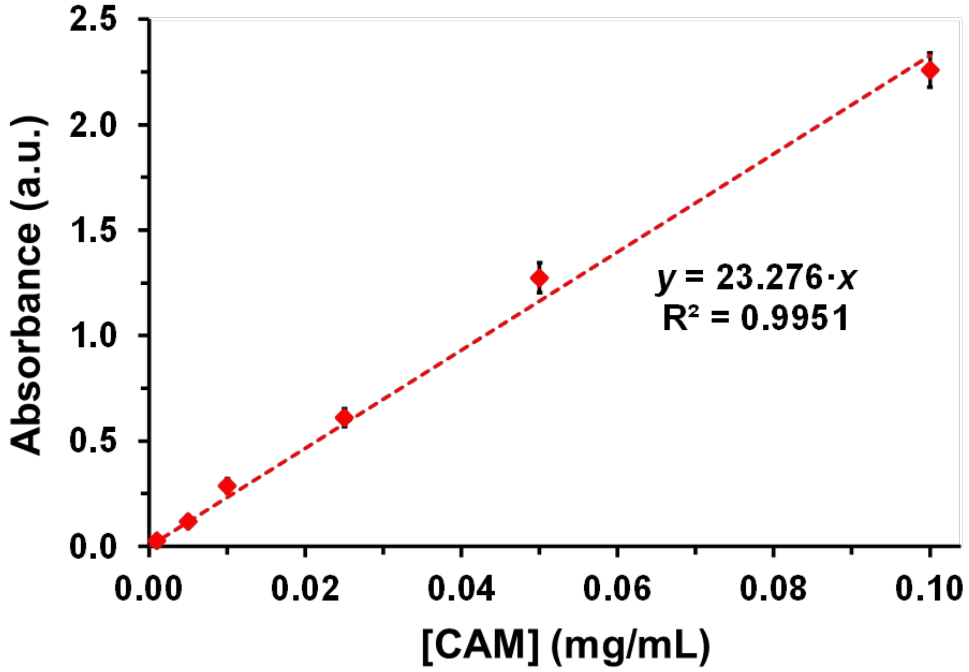

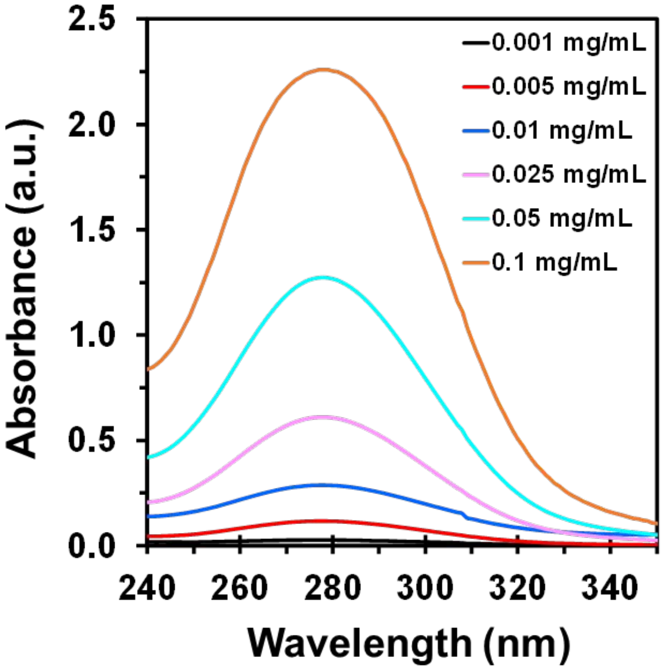

Figure S3. Calibration curve (absorbance-CAM concentration plot) for CAM in PBS.

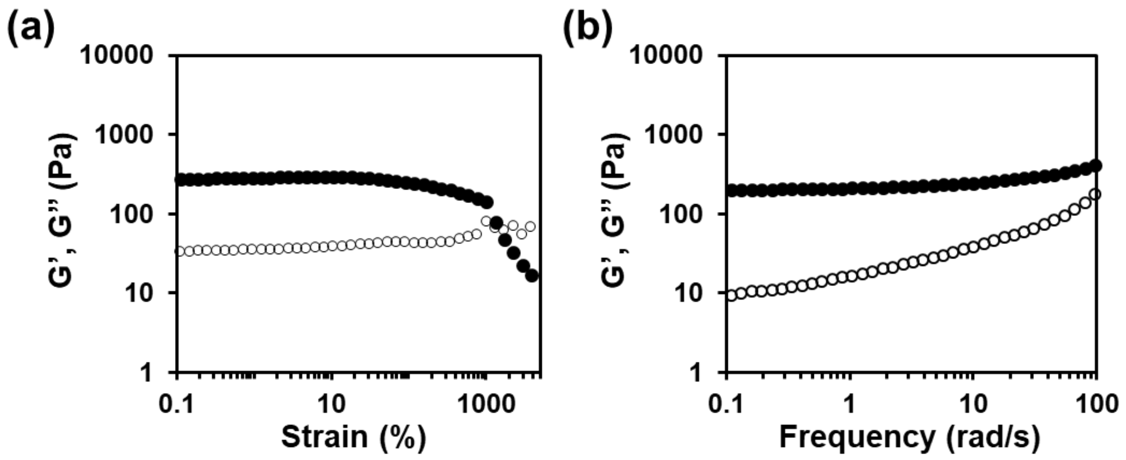

Figure S4. Rheological characterization of as prepared Alg-*g*-PAA/PEDOT/CAM hydrogels. Representative data recorded under (a) amplitude sweep (at 1 Hz) and (b) frequency sweep (strain at 1%) for samples as prepared.
